# Bilateral Pneumonia and Emphysematous Pyelonephritis as an Inaugural Presentation of Diabetes: A Case Report and Review of Literature

**DOI:** 10.7759/cureus.20766

**Published:** 2021-12-27

**Authors:** Murtadha Al-Shaye, Mohammed Elkhazendar, Mustafa Al-Badra, Salah El Rai

**Affiliations:** 1 Department of Clinical Sciences, College of Medicine, University of Sharjah, Sharjah, ARE; 2 Department of Radiology, Sheikh Khalifa Medical City, Ajman, ARE; 3 Department of Radiology, University Hospital Sharjah, Sharjah, ARE

**Keywords:** diabetes complications, pyelonephritis, klebsiella pneumonia, sepsis, diabetes, pneumonia, emphysematous pyelonephritis

## Abstract

We present a case of a 60-year-old male who presented with fever, shortness of breath, left upper quadrant pain accompanied by rigors and chills with a two-week history of productive cough. He had left upper quadrant tenderness and bilateral chest crepitations. The patient became tachypneic, dyspneic, and rapidly progressed to septic shock. Chest x-ray findings of bilateral pulmonary infiltrates on admission were not correlating with the severity of his clinical picture, and blood glucose levels were very high despite a negative prior history of diabetes. Abdominopelvic computed tomography (CT) scans revealed left-sided emphysematous pyelonephritis, which was promptly managed by intravenous antibiotics and CT-guided percutaneous drainage, in addition to glycemic control. This was followed by clinical improvement and resolution of the sepsis. This case sheds light on a possible life-threatening manifestation of the hematogenous spread of pneumonia in uncontrolled diabetic patients, and can even be a de novo presentation of diabetes.

## Introduction

Emphysematous Pyelonephritis (EP) is a rare, life-threatening infection that can rapidly lead to renal parenchymal necrosis, acute deterioration of renal function, and septic shock [1,2]. It usually occurs in a particular subset of patients with either uncontrolled diabetes mellitus or immunocompromised status and requires prompt diagnosis, urgent resuscitation, and timely intervention [1,3,4]. The same category of patients is also susceptible to other infections, including pneumonia, urinary tract infections (UTIs), and soft tissue infections. This poses several challenges in the diagnosis and management of atypical and complicated presentations of EP, which can require more aggressive intervention if discovered late. We present a case of bilateral pneumonia with rapid deterioration and sepsis secondary to a concealed EP. There has been no previous report of EP manifesting as a masked complication of pneumonia. Furthermore, his diabetic status was not known at the time of presentation. Therefore, we would like to highlight the possible life-threatening manifestation of the hematogenous spread of pneumonia in uncontrolled diabetic patients.

## Case presentation

**Figure 1:**
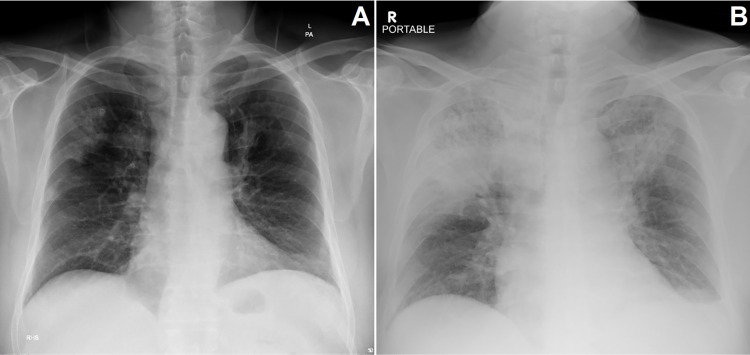
Chest x-ray: (A) posteroanterior view on the first day of admission; (B) anteroposterior view portable on the sixth day of admission The chest x-ray on presentation (A) shows limited and ill-defined patchy peripheral consolidations seen in the right upper and middle zones. There are increased bronchovascular markings bilaterally. No pleural effusion is seen. Mild elevation of the cupola is noted. The follow-up chest x-ray performed six days later in the ICU (B) demonstrates bilateral extensive upper and middle zone consolidations predominantly in the right lung with reduced lucency of the remaining lung fields. Blunting of the left costophrenic angle is observed.

A 60-year-old male presented to the emergency department with a history of fever, sore throat, shortness of breath, and dull left upper quadrant abdominal pain for two days. The pain was non-radiating, relieved by leaning forward and aggravated by lying supine. He also had rigors, chills, nausea, vomiting, and headache. This was preceded by a two-week history of productive cough with whitish sputum. He had no history of chest pain or palpitations, and no urinary frequency, urgency, dysuria, or changed bowel habits. He had a 50 pack/year smoking history and occasional alcohol consumption. Medical history was unremarkable, with no known chronic illnesses and no medications. On admission, he was vitally stable, and physical examination revealed dullness at the right upper lung field with bronchial breath sounds. Abdominal examination revealed left upper quadrant tenderness with no guarding or rigidity, no organomegaly, and no stigmata of liver disease. Cardiac examination was unremarkable. Laboratory investigations (See Appendix) revealed marked leukocytosis, thrombocytopenia, elevated C-reactive protein (CRP) and procalcitonin, and serum glucose of 33.2 mmol/dl. Urine analysis showed 8-10 RBCs, 3-5 WBCs, with negative leukocyte esterase and nitrites; therefore a urine culture was not sent. Chest x-ray showed bilateral peripheral patchy consolidation areas at the right upper and mid lung zones (Figure 1A). Severe acute respiratory syndrome coronavirus 2 (SARS-CoV-2) polymerase chain reaction (PCR) was negative. Oral clarithromycin and IV ceftriaxone were started empirically for bacterial pneumonia, and he was put on an insulin sliding scale.

On the following day, he became distressed with marked tachypnea and Kussmaul breathing. His extremities were cold with weak pulses. He was afebrile but had a heart rate of 118 bpm, respiratory rate of 22/min with labored breathing, blood pressure of 90/66 mmHg, and oxygen saturation of 95%. Blood gases revealed high anion gap metabolic acidosis, hyponatremia, and his serum creatinine level was elevated. IV fluid resuscitation was initiated with oxygen supplementation and isonatric dextrose bicarbonate drip for nephroprotection, and the antibiotics were switched to meropenem. The clinical picture, along with the chest x-ray findings, raised suspicion of an extra-pulmonary septic focus or pulmonary embolism, thus warranting a chest and abdomen CT scan with contrast.

**Figure 2:**
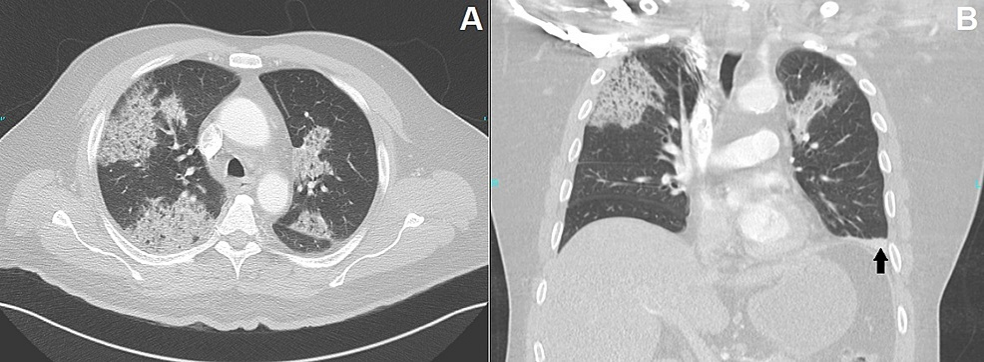
Axial (A) and coronal (B) enhanced chest CT slices in lung window Patchy consolidation areas can be seen in both upper lung lobes. Additionally, there is a subpleural atelectatic change seen in the left lung base (Black arrow in B).

**Figure 3:**
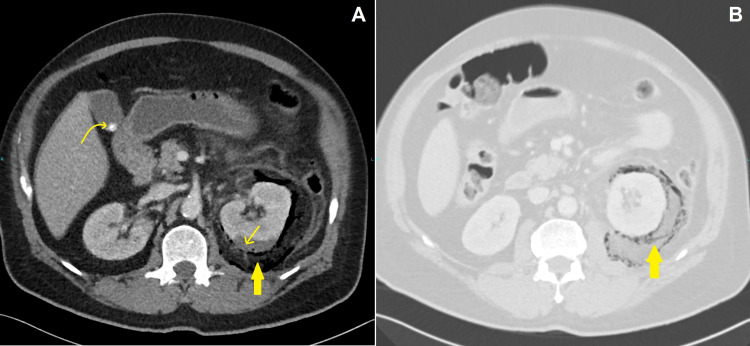
Enhanced abdominal CT axial slices in soft tissue window (A) and lung window (B) of the mid renal poles during the nephrogenic phase A well-organized subcapsular mixed gas-fluid collection in the posterior aspect of the left renal pole is seen (Thin arrow) associated with ipsilateral perinephric gas densities (Thick arrow) comparable to the intraluminal colonic and extracorporeal gas, with thickening of Gerota's fascia seen in (A). It is noteworthy to acknowledge the good renal parenchymal enhancement in the nephrogenic phase. Note the incidental finding of an uncomplicated calcified gallstone (Curved arrow).

**Figure 4:**
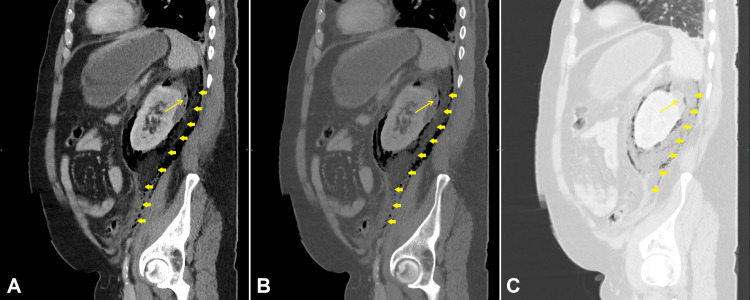
Sagittal reconstructions of enhanced abdominal CT in soft tissue (A), bone (B), and lung (C) windows centered on the left lung Subcapsular fluid is seen in the posterosuperior aspect of the left upper renal pole (Thin arrows) with a large crescent-shaped bubbly perinephric collection extending downwards in the retroperitoneal space along the psoas muscle, reaching the presacral space (Thick arrows). Thickening of Gerota's fascia is seen, which is consistent with emphysematous pyelonephritis.

**Figure 5:**
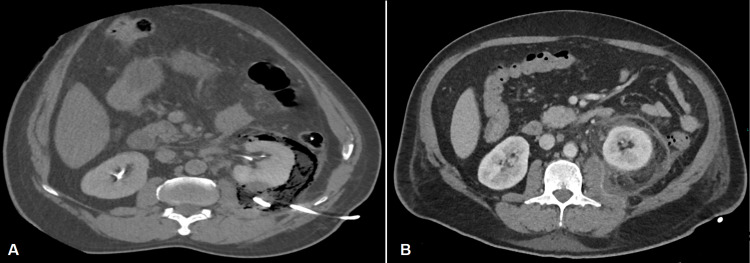
Enhanced abdominal CT axial slices at the left mid renal pole on the second (A) and eleventh (A) days of admission showing the progression of the perinephric collection after catheter drainage An accurately placed pigtail drainage catheter is seen (A) in the left posterior perinephric space at the lower margins of the subcapsular mixed collection and perinephric gaseous collection of type IIIB as per the Huang and Tseng classification. A follow-up image (B) acquired on day 10 post-drainage (i.e. day 11 post-admission) demonstrates persistent Gerota's fascia thickening with perinephric stranding and collection, however, without bubbly or linear streaks of gas.

Vis-à-vis this clinical scenario, a CT scan of chest and abdomen with IV contrast were done, revealing bilateral pulmonary consolidation in the upper lung lobes with subpleural atelectasis in the left lower lobe (Figures 2A-2A) and no pulmonary artery filling defect, thereby excluding pulmonary embolism (not shown here). Furthermore, a left renal subcapsular fluid collection was seen with a gaseous crescent-shaped perinephric collection extending inferiorly in the retroperitoneal space with thickening of Gerota’s fascia (Figures 3A, 3B, 4A, 4B, 4C), consistent with EP. The emphysematous collection was drained under CT guidance with a 12 French pigtail catheter (Figures 5A-5B). The turbid fluid was sent for culture and analysis. After drainage, vancomycin was added, and the patient was shifted to ICU for close monitoring. Culture results of sputum, blood, and the aspirated perinephric fluid yielded *Klebsiella pneumoniae*. A subsequent chest x-ray showed progression of bilateral pulmonary consolidation (Figure 1B), yet the patient’s clinical status improved with resolution of leukocytosis and fever. The patient was later transferred to the ward after seven days in the ICU, with appropriate glycemic control on metformin, and was discharged after complete clinical improvement. He was seen in the endocrine clinic a week later; his HbA1c was 10.7, which emphasizes his previous poor glycemic control.

## Discussion

EP is a rare, potentially life-threatening necrotizing infection of the renal parenchyma and surrounding tissue with characteristic gas formation. It is associated with a high mortality rate due to sepsis and associated multi-organ dysfunction if left untreated [1,2,5]. It has a tendency to affect more females than males and the majority of cases, up to 95%, are associated with underlying uncontrolled diabetes mellitus, which was not only described in known cases of diabetes but also as de novo presentations of long-standing diabetes. Obstruction of the urinary collecting system may also be an underlying cause [1-3]. Pathogens that have been implicated in EP are most commonly *Escherichia coli* (~67% of cases), *K. pneumoniae* (~20% of cases), *Proteus mirabilis*, and coagulase‐negative *Staphylococcus* [1,3,4,6]. The postulated pathogenesis in uncontrolled diabetic patients involves the interplay between hyperglycemia, impaired tissue perfusion, presence of gas-forming bacteria, and immunocompromised status [1,3]. Patients usually present with classic symptoms of pyelonephritis, including fever, flank pain, and dysuria; however, in advanced cases, an initial presentation with acute renal failure and shock prompts urgent intervention due to rapid progression and high mortality [3,4,7]. Other clinical findings include hyperglycemia, microscopic or macroscopic hematuria, and severe proteinuria [1,3]. EP carries a higher mortality risk in the presence of hypotension (systolic blood pressure (SBP)<90mmHg), impaired consciousness, elevated serum creatinine, thrombocytopenia, and bilateral disease; however, serum glucose levels were not associated with greater mortality [3,5].

The non-specific clinical presentation of EP may pose a diagnostic challenge, yet with recent advances and more frequent use of imaging modalities, earlier diagnosis of EP has led to drastic reductions in the overall mortality rate from 78% to 18-21% [4,6,8]. Radiological studies are, therefore, imperative to establish the diagnosis of EP. Plain abdominal kidney, ureter, and bladder (KUB) x-ray may reveal renal shadow enlargement, obscured psoas shadow, and mottled gas within the renal fossa or along the paraspinal region. Abdominal KUB ultrasound may demonstrate enlargement of the kidney with coarse echogenic foci followed by dirty post-acoustic shadowing within the renal parenchyma, collecting system, or perirenal fossa. Yet ultrasound remains operator dependent and non-specific since the gas appearing as echogenic foci may be mistaken for bowel gas or calculi. These imaging modalities were shown to be accurate only in 65% and 69% of cases respectively, compared to 100% accuracy of CT [6,9]. Thus, CT is the imaging modality of choice for diagnosing and evaluating patients with EP due to its high sensitivity and specificity, allowing for the accurate definition of the type and classification of EP gas extension, which guides optimal management and treatment plan [3,4,6]. Contrast medium enhancement may reveal delayed enhancement due to impaired perfusion, and foci of necrosis or abscesses can be seen during the nephrographic phase [9]. CT imaging is also useful to identify predisposing factors for complicated UTIs, such as structural or functional urinary tract abnormalities, including nephrolithiasis, urolithiasis, urothelial neoplasms, and prostate pathologies [9]. It is also useful to rule out other mimics, including but not limited to abdominal abscess and pancreatitis.

There have been two CT-based classification systems used to predict the severity and prognosis of EP as well as guide the management option. Wan Et al. have classified it based on the gas distribution into type 1 EP, characterized by destruction of the renal parenchyma with a mottled or streaky gas pattern and absence of fluid, and type 2 EP, characterized by intrarenal or perirenal fluid collection with a bubbly, loculated gas or presence of gas within the pelvicalyceal system [8]. Type 1 EP has been associated with a more rapid and aggressive clinical course with greater mortality rates [3,5]. Huang and Tseng later on proposed a modified radiological classification system categorizing EP into four classes. In type I, gas is confined to the collecting ducts, in type II, gas is seen within renal parenchyma, in type IIIA, gas extends to the perinephric space, in type IIIB, gas further extends into the pararenal space, and type IV is when EP is bilateral or occurs in a solitary kidney [3].

The initial treatment strategy should start with aggressive fluid resuscitation, oxygen support, correction of acid-base and electrolyte imbalances, initiation of appropriate antibiotic therapy, and glycemic control. Antibiotic therapy should first provide broad-spectrum coverage for gram-negative bacteria and later on be tailored according to the patient’s condition and culture results [1,7]. The subsequent treatment option of EP should be guided by radiological classification as well as the risk factors mentioned above. Historically, emergent nephrectomy was considered the standard of care due to high mortality experienced with conservative management. However, with advancements in interventional radiology, image-guided percutaneous drainage (PCD), in combination with medical management (MM), has yielded greater success in recent years [6-8]. This is particularly beneficial as a kidney preserving alternative to surgery, avoiding the possible morbidities that could occur in patients with a solitary kidney, bilateral EP, or preexisting renal impairment [3]. The available options are MM only, MM plus PCD, MM plus emergent nephrectomy, and MM plus PCD followed by emergent nephrectomy [1, 6]. Recent data has shown conservative management alone to be successful in grades 1 and 2, yet has been associated with increased mortality and warranting further intervention in grades 3 and 4; whereas MM plus PCD has had the lowest mortality rates, as low as 13.5% (higher survival rates compared to emergent nephrectomy only) [4-7]. Nephron-sparing MM plus PCD has shown better outcomes when initiated earlier, even in the context of multiple comorbidities [10]. Yet, nephrectomy may be required in non-responding cases after PCD or after subsequent follow-up CT [4,6,7].

Our case presented with a severe clinical picture of septic shock, and in contrast to the classical presentation of EP, the bilateral pulmonary findings masked the underlying cause of his rapidly deteriorating and non-responding condition. Given the preceding history and the rapid nature of EP progression, it is likely postulated that the subsequent sepsis from pneumonia in this diabetic patient has been complicated by the hematogenous spread to the kidney and resultant rapid progression of EP. This is further supported by isolated culture findings in sputum, blood, and drained fluid of the organism *K. pneumoniae*. The negative urine dipstick on admission, absence of urinary symptoms, and lack of urolithiasis or known prior uropathy go against the possibility of a primarily ascending UTI or asymptomatic bacteriuria. Based on the radiological findings, it is highly unlikely that bilateral pneumonia originated hematogenously from a primary kidney source as it would have led to characteristic radiologic pulmonary findings including the feeding vessel sign, peripheral wedge-shaped opacities, peripheral nodules with or without cavitation, lung abscesses, halo sign, patchy ground-glass opacities, and associated pleural effusions [11]. In comparison, a previously reported case involving EP concurrent with septic pulmonary emboli had CT findings of cavitary lung nodules supporting the septic origin of the pulmonary emboli; moreover, the reported case was known to have diabetes and had urinary symptoms along with hematuria and pyuria but no respiratory symptoms. Notably, *E. coli* was isolated in urine but not in blood [12]. In contrast, our case was unknown to have diabetes, had primarily respiratory symptoms preceding the abdominal symptoms, and had hematuria with borderline pyuria. In addition, *K. pneumonia* was isolated in the sputum, blood, and aspirated perinephric fluid of our case. These differences highlight the rarity of our case and the different mechanisms involved in the concurrent pulmonary and renal findings. This provides a new perspective on the possible complications of pneumonia in uncontrolled diabetic patients. The subtype of EP in our case was IIIB, which responded appropriately to MM and CT-guided PCD. To our knowledge, no previous case has been reported in the literature describing EP as an advanced complication of pneumonia in uncontrolled diabetes.

## Conclusions

The hyperglycemic hypoperfused tissue microenvironment in uncontrolled diabetes facilitates the development of emphysematous infections, reminding us of the importance of diabetes screening and primary prevention before such complications occur. As shown in this case, EP can potentially be a life-threatening consequence of the hematogenous spread of pneumonia in uncontrolled diabetic patients, which can be missed in cases with undiagnosed diabetes. Furthermore, a negative urine dipstick or culture may not exclude EP in the presence of sepsis. Therefore, earlier imaging, specifically with CT scan due to its high sensitivity and specificity, can establish the diagnosis and guide the optimal management based on the classification of EP. With earlier diagnosis and further advancements in interventional radiology, minimally invasive percutaneous drainage combined with medical management has better outcomes and a nephron-sparing potential compared to nephrectomy.

## Appendices

**Table 1:** Laboratory investigations done during the hospital stay RBC, red blood cell; MCV, mean cell volume; MCH, mean cell hemoglobin; MCHC, mean cell hemoglobin concentration; WBC, white blood cell; ALP, alkaline phosphatase; ALT, alanine aminotransferase; CRP, c-reactive protein; PT, prothrombin time; PTT, partial thromboplastin time; INR, international normalized ratio; AFB, acid-fast bacilli; SARS-CoV-2, severe acute respiratory syndrome coronavirus 2; PCR, polymerase chain reaction; Na, Sodium; K, Potassium; Cl, Chlorine; CO2, Carbon dioxide; CBC: complete blood count; O^2^, Oxygen; HCO_3_, Bicarbonate;

Investigation	Value	Normal Value
CBC		
RBC	4.94 x 10^6^/µL	4.35 - 5.65 x 10^6^/µL
Hemoglobin	16.6 g/dL	13.6 - 16.9 g/dL
Hematocrit	46.90 %	38.3 - 48.60 %
MCV	94.9 fL	80.0 - 100.0 fL
MCH	33.6 pg (high)	27.00 - 32.00 pg
MCHC	35.40 g/dL	32.00 - 36.00 g/dL
Platelets	95 x 10^3^/µL (low)	150 - 450 x 10^3^/µL
WBC	23.3 x 10^3^/µL (high)	4.0 - 11.0 x 10^3^/µL
Neutrophils	86 % (high)	40 - 80 %
Lymphocyte	4.90 % (low)	18.00 - 42.00 %
Monocyte	9.00 %	2.00 - 11.00 %
Eosinophil	0.00 % (low)	1.00 - 6.00 %
Basophil	0.10 %	0.00 - 2.00 %
Chemistry		
Glucose	33.2 mmol/L (high)	4.1 - 5.5 mmol/L
Na^+^	129 mmol/L (low)	136 - 145 mmol/L
K^+^	4.56 mmol/L	3.50 - 5.10 mmol/L
Cl^-^	89.3 mmol/L (low)	98 - 107 mmol/L
CO_2_	12.6 mmol/L (low)	23.0 - 30.0 mmol/L
Anion Gap	27 mEq/L (high)	8 - 12 mEq/L
Urea level	14.4 mmol/L (high)	2.10 - 7.10 mmol/L
Creatinine	250 µmol/L (high)	62 - 115 µmol/L
Albumin	29.1 g/L (low)	34.0 - 50.0 g/L
Total Protein	71 g/L	64 - 82 g/L
ALP	53 IU/L	46 - 116 IU/L
ALT	23 IU/L	16 - 63 IU/L
Total Bilirubin	32.3 µmol/L (high)	3.0 - 17.0 µmol/L
Lactic Acid	4.2 mmol/L (high)	0.4 - 2.0 mmol/L
Acute Phase Reactants		
CRP	152.15 mg/L (high)	0.0 - 3.0 mg/L
Procalcitonin	>100.000 µg/L (high)	0.00 - 0.05 µg/L
PT/PTT/INR		
PT	22.40 sec (high)	9.00 - 14.00 sec
PTT	44.80 sec (high)	25.00 - 35.00 sec
INR	1.71 (high)	0.80 - 1.29
Arterial Blood Gases (ABG)		
pH	7.27 (low)	7.35 7.45
pCO_2_	16.6 mmHg (low)	35.0 - 45.00 mmHg
pO_2_	84 mmHg	80 - 100 mmHg
O_2 _Saturation	95.50%	95.00 - 100.00 %
HCO_3_	12.9 mEq/L (low)	22 - 28 mEq/L
Anion Gap	19.5 mEq/L (high)	8.0 - 12.0 mEq/L
Urine Analysis		
Appearance	Red	Yellow
pH	5 (low)	4.6 - 8.0
Protein	Negative	Negative
Glucose	Negative	Negative
Bilirubin	Negative	Negative
Urobilinogen	0.2 mg/dl	0.2 - 1.0 mg/dL
Ketones	Negative	Negative
Nitrites	Negative	Negative
Leukocyte esterase	Negative	Negative
WBC	3-5 /hpf	0-5 /hpf
RBC	8-10 /hpf (high)	0-4 /hpf
Squamous Epithelial Cells	Few	Absent
Microbiology & Cultures		
Blood	Klebsiella pneumoniae	
Sputum	Klebsiella pneumoniae	
Sputum AFB	Negative	
SARS-CoV-2 PCR	Negative	
Drained Fluid	Klebsiella pneumoniae	
